# pH-Responsive Host–Guest Complexation in Pillar[6]arene-Containing Polyelectrolyte Multilayer Films

**DOI:** 10.3390/polym9120719

**Published:** 2017-12-16

**Authors:** Henning Nicolas, Bin Yuan, Jiangfei Xu, Xi Zhang, Monika Schönhoff

**Affiliations:** 1 University of Muenster, Institute of Physical Chemistry, Correnstraße 28/30, 48149 Münster, Germany; h_nico01@uni-muenster.de; 2 Key Laboratory of Organic Optoelectronics & Molecular Engineering, Department of Chemistry, Tsinghua University, Beijing 100084, China; shibingriji@163.com (B.Y.); xujf@mail.tsinghua.edu.cn (J.X.); xi@mail.tsinghua.edu.cn (X.Z.)

**Keywords:** host-guest chemistry, layer-by-layer self-assembly, pillar[6]arene, pH-responsiveness

## Abstract

A water-soluble, anionic pillar[6]arene derivative (WP6) is applied as monomeric building block for the layer-by-layer self-assembly of thin polyelectrolyte multilayer films, and its pH-dependent host–guest properties are employed for the reversible binding and release of a methylviologen guest molecule. The alternating assembly of anionic WP6 and cationic diazo resin (DAR) is monitored in-situ by a dissipative quartz crystal microbalance (QCM-D). In solution, the formation of a stoichiometric inclusion complex of WP6 and cationic methylviologen (MV) as guest molecule is investigated by isothermal titration calorimetry and UV-vis spectroscopy, respectively, and attributed to electrostatic interactions as primary driving force of the host–guest complexation. Exposure of WP6-containing multilayers to MV solution reveals a significant decrease of the resonance frequency, confirming MV binding. Subsequent release is achieved by pH lowering, decreasing the host–guest interactions. The dissociation of the host–guest complex, release of the guest from the film, as well as full reversibility of the binding event are identified by QCM-D. In addition, UV-vis data quantify the surface coverage of the guest molecule in the film after loading and release, respectively. These findings establish the pH-responsiveness of WP6 as a novel external stimulus for the reversible guest molecule recognition in thin films.

## 1. Introduction

The unique host–guest chemistry of macrocyclic molecules has experienced tremendous attention in the past years and promoted intensive scientific efforts in the fields of surface chemistry, polymer research, and material science. A variety of host–guest inclusion complexes, supramolecular polymers, and self-assembled nanostructures based on the stimuli-responsive properties of host molecules as the key recognition component were developed [1,2,3,4]. In continuation of a series of well-established hosts such as cyclodextrins [4,5,6,7,8,9] or cucurbiturils [10,11,12,13,14,15], pillar[*n*]arenes (P[*n*]) are rather novel representatives as they were only recently reported by Ogoshi et al. in 2008 [16,17,18,19]. The Lewis-acid catalyzed condensation of 1,4-dimethoxybenzene or hydroquinone units, respectively, yielded pillar[5]arene derivatives bridged by methylene groups in their 2- and 5-positions, therefore revealing a macrocyclic structure with a highly symmetrical shape. Since then, this novel type of host has attracted great attention on account of the ease of chemical modification, resulting from the reactive substituents located at the upper and lower rim, respectively. Due to the selective addressability of the reactive groups, a series of homologues (*n* = 5–15) bearing a large variety of properties are available to date, although the synthesis of higher homologues still suffers from low yields [20,21]. The π-electron-rich cavity combined with the opportunity of structure-tailoring motivated scientists to investigate the potential of P[*n*]s in the field of host–guest chemistry. The molecular recognition of P[*n* = 5, 6] towards a series of guest molecules has been reported, showing that cooperative host–guest-interactions such as electrostatics, hydrophobic interactions, charge transfer interactions, hydrogen bonding, and π-π-stacking are responsible for driving the complexation [22,23]. In addition, the introduction of stimuli-responsiveness on either the guest or the host enabled the fabrication of P[*n*]-guest based complexes, receptors, and drug delivery systems whose assembly could reversibly be switched by tuning either a single or multiple stimuli such as pH, light irradiation, or redox conditions [24,25,26,27,28,29,30,31].

Surface molecular imprinted polyelectrolyte multilayer films (SmiLbL films) are based on the layer-by-layer self-assembly of oppositely charged polyelectrolytes [32,33,34,35] and have opened an established class of thin and structured nanomaterial that bears imprinted binding sites for the reversible binding and release of small organic molecules [36]. In such polymer films, the binding sites are located comparatively close to a surface, therefore solving the problem of long diffusion pathways and buried binding sites known from macroscopic molecular imprinted materials [37,38]. Shi et al. prepared the first SmiLbL film in 2007 by employing a macromolecular complex first fabricated from both (Poly)acrylic acid and a porphyrin derivative, and then used it as building block for the LbL preparation of a polyelectrolyte multilayer film [39]. This initial SmiLbL approach was based on a pH-tunable electrostatic interaction to yield quantitative and reversible uptake and release [40,41]. Similar concepts aim at an optimization of selectivity by employing more specific interactions for molecular recognition, such as disulfide [42] or amide bonds [43] charge transfer interactions [44] and hydrogen bonds [45,46].

However, since the soft and flexible nature of such films allows swelling and structural deformation of the imprinted binding sites, their performance suffered in terms of reversibility and binding efficiency. For this reason, we recently applied a molecular host, cucurbit[8]uril (CB[8]), as artificial binding site within a polyelectrolyte multilayer film in order to take advantage of the intrinsic structural rigidity. It was found that, although swelling of the film was still observed, CB[8] was indeed unaffected from the film dynamics, i.e., binding site deformation was successfully avoided. Therefore, the host–guest chemistry of CB[8] could be implemented in polyelectrolyte multilayer films and be able to perform the reversible binding and release of a guest molecule controlled by either redox-conditions [47] or light irradiation [48]. A quite high binding efficiency over several binding and release cycles was observed [49]. Furthermore, the initial imprinting step, realized via pre-complexation during preparation, was no longer required as a result of the ready-made character of CB[8] as artificial binding site. Similarly, cyclodextrin hosts have more recently been employed in LbL films to achieve reversible, triggered guest uptake and release [50,51,52].

More recently, we have also employed a monomeric and water-soluble pillar[6]arene derivative (WP6) as negatively charged building block for the layer-by-layer self-assembly of a multilayer film, which bears anionic layers solely consisting of host molecules [53]. A proof of principle of a host–guest inclusion complex formation was provided by exposing the host-containing multilayer films to a viologen guest molecule. After complexation, release was achieved by washing off the guest with the help of a solvent change, thus demonstrating the full reversibility of the binding event in the film. In this way, the pre-association of the host with a polyelectrolyte, as it was previously required in order to incorporate CB[8] into the multilayer films, was no longer required and therefore the volume of inoperative polymer material in such novel WP6-containing films was drastically reduced.

In the present work, we have developed this approach further, as we introduce a new trigger to this optimized release system and report on the pH value as an external stimulus that enables precise control over the alternating binding and release processes of a viologen guest in WP6-containing multilayer films.

**Figure 1:**
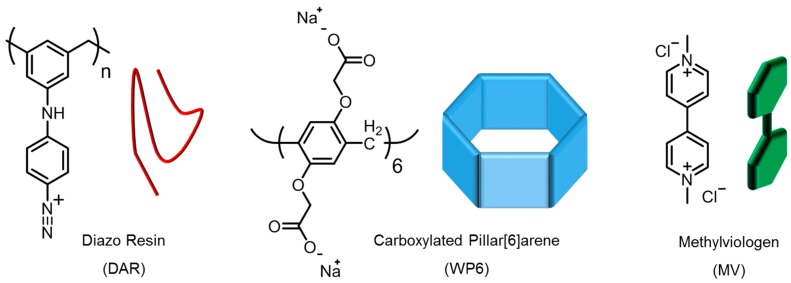
Chemical structures of the building blocks diazo resin polymer (DAR) and carboxylated pillar[6]arene (WP6), as well as the guest molecule methylviologen dichloride (MV).

**Figure 2:**
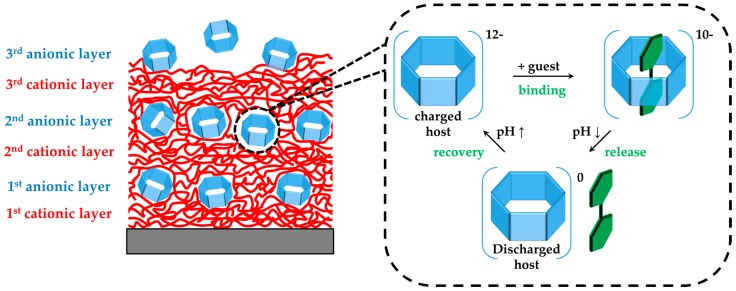
Sketch of the multilayers formed of cationic diazo resin polymer (DAR) and anionic carboxylated pillar[6]arene (WP6), indicating the mechanism of binding and release of the guest molecule methylviologen dichloride (MV). Net charges of the complex are ideal values, neglecting the charge loss upon photo-crosslinking.

We engaged the pH sensitivity of the carboxylated rims of WP6 (see Figure 1) in order to manipulate the electrostatic interactions between host and guest, and therefore control the binding by de-/protonation, respectively. For this purpose, the pH-dependent host–guest complexation was first investigated in solution by employing UV-vis and isothermal titration calorimetry (ITC) experiments. Then, a dissipative quartz crystal microbalance (QCM-D) was employed in order to monitor in-situ the LbL self-assembly of multilayer films (DAR/WP6)_30_ obtained from the alternating deposition of both the photosensitive polycation diazo resin (DAR) and WP6 (see Figure 1, left). The multilayer films were then crosslinked by photo-induced formation of covalent ester bonds between the decomposed azido groups of DAR and carboxylate groups of WP6 [54]. Subsequently, the pH-dependence of the host–guest complex formation is investigated by first loading the guest molecule into the film at basic conditions and then releasing it by protonation of the host molecule as a result of pH lowering; see schematic illustration in Figure 2. In addition, we prepared the same multilayer films on UV-permeable quartz slides and addressed the quantification of the guest molecule surface coverage (Γ_MV_) by employing UV-vis spectroscopy.

## 2. Materials and Methods 

### 2.1. Materials

Sodium acetate, sodium dihydrogen phosphate, and disodium hydrogen phosphate were obtained from Merck (Kenilworth, NJ, USA). Acetic acid was purchased from VWR International (Radnor, PA, USA). Methylviologen dichloride hydrate (98%) and diluted solutions of NH_3_ (25%) and H_2_O_2_ (30%) were provided by Sigma Aldrich. DAR and WP6 (see structures in Figure 1) were synthesized as described before [53]. All compounds were used as received.

### 2.2. Solutions

All compounds were prepared in buffer solutions of different pH values. Phosphate buffer (pH = 7.8) and acetate buffer (pH = 4.8 or 3.6) were prepared in ultrapure water (Merck Millipore, Darmstadt, Germany, resistivity > 18 MΩ∙cm) with a concentration of both 10 mmol L^−1^ and 50 mmol L^−1^, respectively. Solutions of MV (1 mmol L^−1^) and WP6 (0.1 mmol L^−1^) were prepared in both phosphate buffer (50 mmol L^−1^) and acetate buffer (50 mmol L^−1^), and used for ITC experiments. For multilayer film preparation, DAR (0.1 mmol L^−1^) and WP6 (0.1 mmol L^−1^) solutions, respectively, were prepared in 10 mM phosphate buffer (pH 7.8). Solutions for UV-vis experiments were diluted from such stock solutions.

### 2.3. Characterization of the MV–WP6 Complex

The thermodynamic parameters of the host–guest complexation between MV (1 mmol L^−1^) and WP6 (0.1 mmol L^−1^) were investigated at different pH values (pH = 7.8 or 4.8) by isothermal titration calorimetry. The solutions were degassed for 15 min and the experiments were then performed on a Nano ITC (TA Instruments, New Castle, DE, USA). MV solution (250 μL) was titrated into WP6 solution (953 μL). The successively titrated volume was 10 μL and injected in intervals of 5 min. The obtained data was evaluated by using the software “NanoAnalyze” (Version 3.3.0, TA Instruments, New Castle, DE, USA, 2005/2014).

The host–guest complexation was further investigated by UV-vis spectroscopy (UV-2550 photometer, Shimadzu, Kyoto, Japan). The absorbance spectrum of MV (0.05 mmol L^−1^, in 10 mM phosphate buffer, pH 7.8) was recorded by using pure solvent as reference. Then, a stoichiometric amount of WP6 was added to the MV solution in order to fabricate a 1:1 inclusion complex. The absorbance spectrum of the mixture was taken and referenced to an equimolar solution of WP6. In this way, solely the contribution of MV after complexation was recorded.

### 2.4. Substrate Cleaning

Gold-coated quartz sensors (QSX-301, Q-sense, Biolin Scientific, Gothenburg, Sweden) with a resonance frequency of f_0_ = 4.95 MHz were used to study both the multilayer film formation and the subsequent guest molecule binding and release processes. Before use, the sensors were heated in RCA solution (NH_3_/H_2_O_2_/H_2_O = 1:1:5) for 20 min at 70 °C. UV-permeable quartz slides (Hellma Analytics, Mülheim, Germany) were employed as substrates for the preparation of dip-coated multilayer films and previously cleaned using the same procedure.

### 2.5. In Situ Preparation of Multilayer Films and Monitoring by QCM-D

Layer-by-layer self-assembled multilayer films (DAR/WP6)_30_/DAR were prepared by alternating deposition of the building blocks DAR and WP6. The preparation was performed in flow cells allowing in situ dissipative quartz crystal microbalance (QCM-D) experiments in a Q-Sense E4 apparatus (Biolin Scientific, Gothenburg, Sweden). All experimental work involving the use of DAR was carried out in a dark lab in order to avoid the light-induced decomposition of the photosensitive diazo groups. Tubes and flow cell were initially filled with phosphate buffer (pH 7.8) and equilibrated for at least 20 min. The flow rate was 0.1 mL min^−1^ and kept constant throughout all QCM-D experiments. The resonance frequency f_0_ as well as its overtones were then determined several times until a stable baseline (Δf < ± 1 Hz within a period of 10 min) was detected. A programmable autosampler was used to control the alternating flow of solutions and phosphate buffer. DAR solution overflows the sensor for 20 min to form an initial cationic layer. Its completion was indicated by a plateau of the deviation from the baseline, Δf. Subsequently, phosphate buffer was used for 20 min to remove loosely attached polyelectrolytes from the surface. Then, the WP6 solution was flown over the sensor for 10 min in order to adsorb an anionic layer. Finally, the film was overflowed by phosphate buffer again for 10 min. By following this cyclic procedure, (DAR/WP6)_30_/DAR was fabricated. After preparation, the sensor was irradiated by visible light (λ = 440 nm) for 5 min in order to photo-crosslink the multilayer film.

### 2.6. Dip-Coating of Multilayer Films

In order to enable UV-vis investigations of the multilayer films, (DAR/ WP6)_30_/DAR films were prepared via dip-coating by a programmable dipping robot (DR-1, Riegler and Kirstein, Berlin, Germany). Cleaned quartz slides were alternatingly immersed into the respective solutions by the robot, thereby using the same sequence as well as identical adsorption times as for the QCM-D experiments. After each layer deposition, the samples were washed in three individual buffer solutions for 2 min, respectively. The multilayer films were then characterized by UV-vis before and after photo-crosslinking. As reference, an uncoated quartz slide was used.

### 2.7. Guest Molecule Binding and Release

After photo-crosslinking, the coated QCM-D sensor was remounted and phosphate buffer (pH 7.8) was exposed to the sample for 2 h in order to equilibrate the multilayer film with respect to the content of hydration water. Again, resonance frequency and overtones were determined in terms of a stable baseline as described above. The multilayer films were then exposed to a solution of MV (1 mmol L^−1^, pH 7.8) for 3 h. This was followed by flowing acetate buffer (pH 3.6) over the sensor for 1 h in order to induce the guest molecule release. Finally, the acidic buffer was replaced by the phosphate buffer (pH 7.8) to recreate basic conditions. 

## 3. Results

### 3.1. pH-Tunable Host–Guest Chemistry in Aqueous Solution

**Figure 3:**
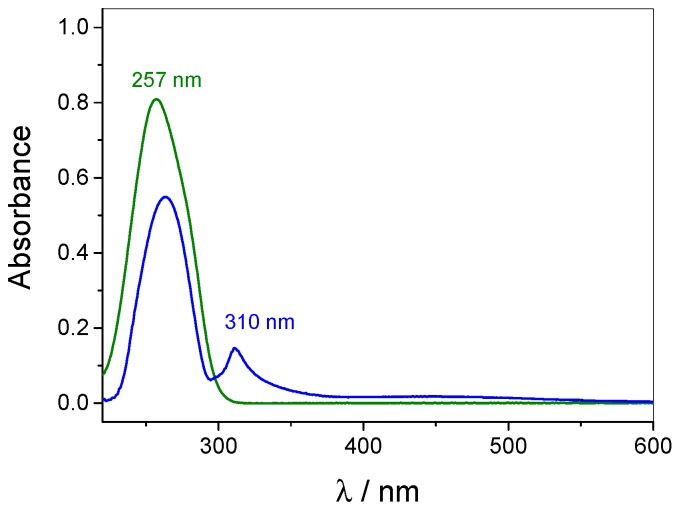
UV-vis spectra of MV solution (50 μmol L^−1^) against water as reference (green line) and MV solution containing an equimolar ratio of WP6 against WP6 solution as reference (blue line).

The host–guest complexation of WP6 and MV is observed by UV-vis spectroscopy. Spectra of free MV (green line in Figure 3) and MV complexed with WP6 (blue line) show a distinct difference, which imply the interaction of the guest with the host. The stoichiometric addition of WP6 to an MV solution significantly reduces the intensity of the absorbance band of the guest at 257 nm and therefore indicates intermolecular interactions between both components. Also, this absorbance maximum is bathochromically shifted by about 6 nm. Furthermore, an additional absorbance band can be observed at around 310 nm. Since WP6 alone bears an absorbance maximum at 292 nm (see Supplementary Figure S1 in the supporting information), the new band might be the result of changed absorbance properties of the host due to the intermolecular interactions with the guest molecule. Additionally, a very weak charge transfer band is detected in the regime between 400 and 550 nm [22]. The latter was even more clearly identified by measuring the complex solution at an increased concentration (see Supplementary Figure S2), thus a charge transfer interaction between the electron-rich cavity and the electron-poor guest molecule is indicated. 

We further employed ITC experiments in order to identify the thermodynamic properties of the host–guest complexation. MV was first titrated into a solution of WP6 at pH 7.8 and a binding constant of k_a_ = (1.53 ± 0.39) 10^6^ M^−1^ was extracted from a single site binding model (see Table 1 and Supplementary Figure S3 for raw data). The determined stoichiometry of *n* = 0.92 ± 0.07 further confirmed that a 1:1 complex was formed, as expected [22]. We then repeated the ITC experiments at pH 4.8 in order to figure out if acidic conditions are able to prevent the host–guest complexation due to the protonation of WP6. ITC data still indicate the formation of a 1:1 complex, however, the binding constant was lowered by about one order of magnitude (see Table 1 and Supplementary Figure S4). We therefore concluded that WP6 is not fully protonated at pH 4.8, and still allows the formation of the inclusion complex due to electrostatic interactions. A reduced number of dissociated carboxyl groups on WP6 at pH 4.8 might also lead to a lower solvent contribution to the entropy and act as the reason for the significantly reduced binding entropy, again indicating a weakened complexation. We intended to perform the experiments at an even lower pH of 3.6, however, WP6 was not soluble at all at this conditions, although the concentration required for ITC is very low. From this observation, we concluded that the charge of the host is sufficiently reduced by such a pH of 3.6 [30]. Thus, the experiments in solutions established pH-dependent conditions suitable for uptake and release of the guest from WP6.

### 3.2. In Situ QCM-D Study of Multilayer Formation

**Figure 4:**
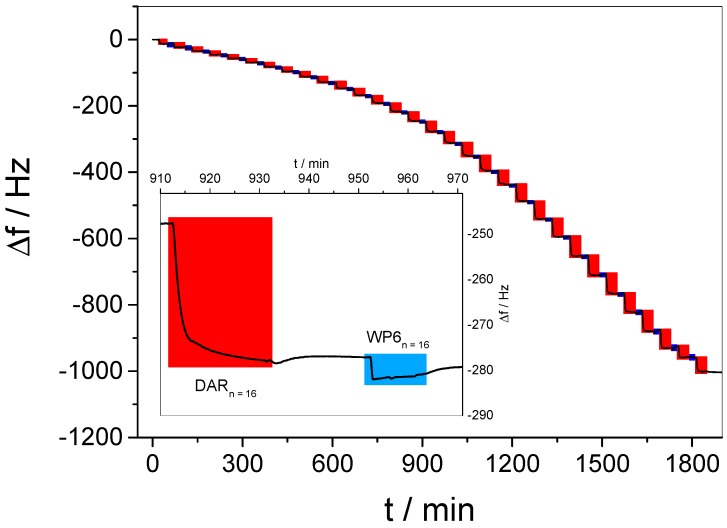
Development of Δf for the Layer-by-Layer (LbL) preparation of a multilayer film (DAR/WP6)_30_/DAR by alternating deposition of DAR (highlighted in red) and WP6 (highlighted in blue), respectively. A close-up of the deposition of a typical bilayer is shown as insert.

A typical time-dependent change of the resonance frequency, Δf, was obtained for the alternating layer-by-layer deposition of DAR and WP6 (see Figure 4). Please note that for clarity, only the fifth overtone of the resonance frequency is shown, the entire ensemble of overtones can be seen in Supplementary Figure S5. For the deposition of each layer, a decrease of the resonance frequency was monitored, thus the formation of a multilayer film (DAR/WP6)_30_/DAR was demonstrated.

**Figure 5:**
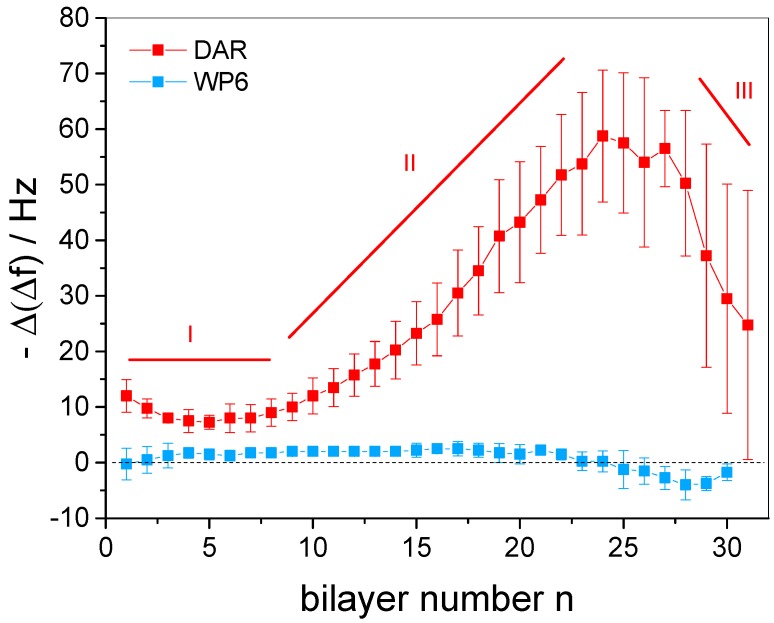
Changes of the resonance frequency for the individual adsorption of a DAR (−Δ(Δf_DAR_), red squares) and a WP6 (−Δ(Δf_WP6_), blue squares) layer during preparation of multilayer films (DAR/WP6)_30_/DAR.

From the insert in Figure 4, it can further be seen that the adsorption of DAR causes a significantly stronger decrease of Δf in comparison to WP6. In order to analyze the growth behavior of the multilayer film, the frequency changes detected per individual layer, −Δ(Δf), were evaluated and given in dependence of the bilayer number *n* in Figure 5. It is obvious that the mass increment per DAR layer can be separated into three individual regimes (see red squares in Figure 5). Initially, the DAR adsorption causes Δf changes per individual DAR layer −Δ(Δf_DAR_) of 8 Hz to 12 Hz (*n* = 1–10). Then, continuously thickened layers are monitored (13 Hz ≤ −Δ(Δf_DAR_) ≤ 59 Hz for *n* = 10–25). Finally, clearly decreasing −Δ(Δf) changes are indicated at the end of the preparation (58 Hz ≥ −Δ(Δf_DAR_) ≥ 25 Hz).

As a potential reason for the different growth regimes, the basic experimental conditions must be taken into account. In order to keep the WP6 permanently deprotonated, all employed sample solutions during film preparation were adjusted to pH 7.8, although DAR is sensitive to basic conditions [55]. To identify the potential decomposition of DAR in basic solution, a reference experiment monitored the time-dependent development of the DAR absorbance (see Supplementary Figure S6). A continuous decrease of the main DAR absorption band was detected, indicating the slow decomposition of the azido functions and giving a plausible explanation for the different growth regimes. This decomposition yields the same spectral changes as the crosslinking, compare Supplementary Figures S6 and S7. Since the cationic diazo groups are essential for the electrostatically driven layer-by-layer assembly of the multilayer film, their slow decomposition causes the need of increasing amounts of DAR in order to accomplish the charge overcompensation of the previous WP6 layer. The mass increment per layer is supported by a softening of the film, as shown by the strongly increasing dissipation values in Supplementary Figure S8. Such additional mass, increasing with layer number, might be represented by −Δ(Δf_DAR_) of the second growth regime. After several hours, the remaining charge density of DAR might be too weak to overcompensate WP6 and therefore leads to thinner DAR layers, resulting in the third growth regime.

In contrast, −Δ(Δf) values observed for the layer formation of WP6 do not suggest different growth regimes (see blue squares in Figure 5). The −Δ(Δf_WP6_) is in the range from −4 Hz to +2 Hz for all *n*, thus indicating a similar content of the host for each anionic layer. Such small values and the occurrence of negative values might, on the one hand, be due to changes of layer hydration upon WP6 adsorption, as the surface coverage of hydration water in the films is included in the detected frequency change. On the other hand, one would expect a much stronger frequency decrease as a consequence of the deposition of a full WP6 monolayer. The obtained data therefore indicate that the WP6 molecules are not deposited as a closely packed monolayer, but are rather located with large intermolecular distances. This might be due to the high charge of WP6, requiring only a low mass coverage for charge overcompensation. On the other hand, strong mutual electrostatic repulsion of WP6 molecules might play a role since it was recently shown that strongly charged gold nanoparticles yield only sub-monolayer coverage in LbL assembly [56].

In addition, the low WP6 amount questions the role of electrostatic interactions regarding the LbL assembly of the multilayer film. A control experiment shed some light into this uncertainty, wherein DAR was initially adsorbed on a QCM sensor, resulting in a typical Δf_DAR_ value of −12 Hz (see Supplementary Figure S9). After washing, DAR was again offered to the surface in order to examine whether the omitted deposition of WP6 influences the multilayer growth. Indeed, only a very slight mass deposition was detected after such procedure was performed twenty times in total (compare Supplementary Figure S9). This reasons the conclusion that the low amounts of deposited WP6 observed during film preparation is fundamentally essential for a regular buildup, i.e., the electrostatic interactions between both building blocks were identified as the driving force of the LbL assembly.

### 3.3. In Situ Investigation of Guest Binding and Release

**Figure 6:**
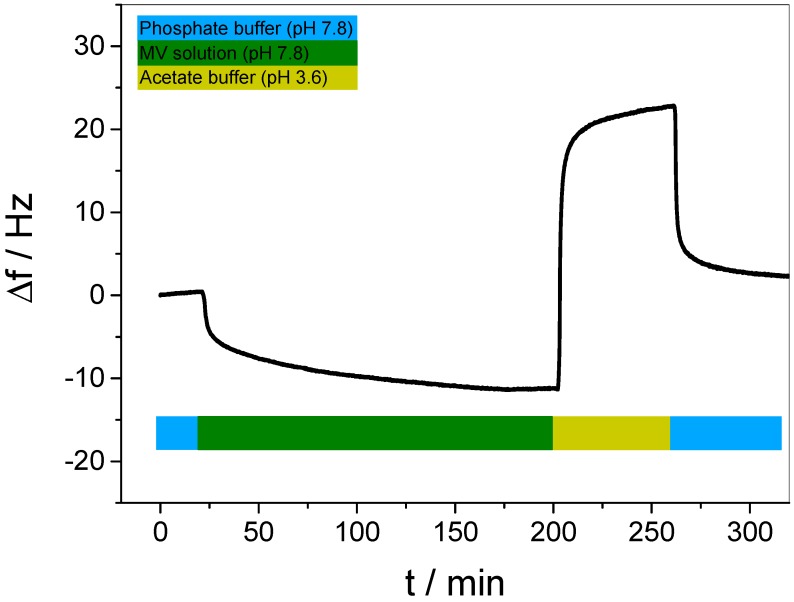
Resonance frequency difference, Δf, upon exposure of a (DAR/WP6)_30_/DAR multilayer film to MV solution (pH 7.8, green) and acetic buffer (pH 3.6) as the releasing agent (yellow). The blue bars represent the exposure of the film to phosphate buffer (pH 7.8).

After photo-crosslinking the multilayer film, the film was exposed to a basic solution of MV (pH 7.8) in order to achieve binding of the guest into the WP6 layers. In QCM-D, a significant decrease of the resonance frequency over a period of 3 h was observed (see Figure 6, highlighted in green), indicating that the guest molecule is incorporated into the multilayer film. The main mass increment occurs within only a few minutes, while a subsequent, lower mass increment rate was detected over several hours, until a plateau was reached, thus indicating the complete saturation of the film.

We note here that the carboxylic groups of WP6 are partly turned into ester bonds due to the crosslinking with DAR, however, as the uptake and release experiments show, a sufficient fraction of carboxylic groups remains unreacted and can thus act as a negatively charged site enabling MV uptake.

In order to release MV, the loaded multilayer film was exposed to acetate buffer (pH 3.6) for 1 h to reduce the attractive electrostatic interaction between host and guest. As a result, the corresponding response of the Δf development reveals a rapid increase that clearly confirms the release of mass from the multilayer film (see Figure 6, highlighted in yellow). However, the second plateau clearly exceeds Δf = 0, therefore indicating that the released amount of mass was significantly higher than the previously incorporated mass. Since QCM-D detects mass changes of all components involved, the occurrence of additional molecular processes besides guest molecule binding and release must be considered as a contribution to the enhanced mass release. The identification of such processes will be discussed later.

**Figure 7:**
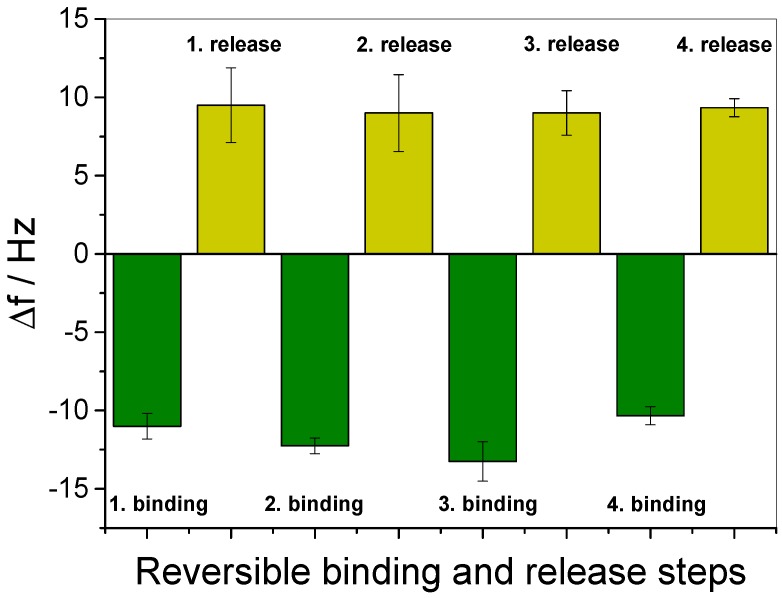
Dissipative quartz crystal microbalance (QCM-D) experiments of binding and release of MV, averaged over four individual (DAR/WP6)_30_/DAR films (green bar: Δf change upon guest molecule binding; yellow bar: Δf change detected after guest molecule release by pH lowering (7.8 to 3.6) and subsequent re-equilibration of the multilayer film back to pH 7.8. Yellow bars thus compensate for hydration changes and include guest molecule mass changes only.

The subsequent reestablishment of the basic conditions by increasing the pH to 7.8 sheds some light into such additional molecular processes. In Figure 6, highlighted in blue, a clear decrease of Δf indicated an increase of mass of the multilayer film, which ends in a third plateau at Δf ≈ +2 Hz, indicating that the multilayer film has returned to the original state before guest binding, thus a complete removal of MV can be concluded. For verification, the pH-dependent binding and release was performed several times by repeating the cycle (see Supplementary Figure S10), revealing a similar Δf development for each cycle. Binding and release data, extracted from the plateau values and averaged over four individual samples, is shown in Figure 7. Please note that the given values for the release (yellow bars in Figure 7) were calculated by subtracting the plateau value of guest saturation from the plateau value after reestablishing the basic conditions (blue highlighted in Figure 6). The data clearly demonstrate that the binding and release of MV in WP6-containing multilayer films is pH-responsive and full reversible.

**Figure 8:**
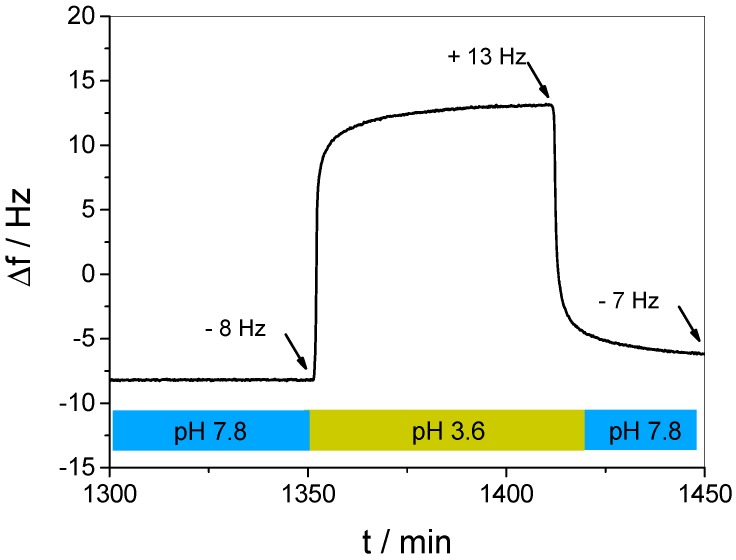
Δf change of a (DAR/WP6)_30_/DAR multilayer film resulting from alternatingly changing the pH from 7.8 (blue) to 3.6 (yellow) without presence of a guest molecule.

It was already mentioned that the loss of mass resulting from the acidic treatment (Figure 6, highlighted in yellow) indicates the simultaneous occurrence of both the release of the guest and other molecular processes in the multilayer film. In order to identify such secondary processes, we employed acidic conditions to a multilayer film after the guest was fully released (see Figure 8).

It is obvious that the alternating pH already causes strong and fully reversible changes in the resonance frequency. This is most likely due to the fact that the pH-induced protonation/deprotonation of WP6 in the film results in a disturbance of the charge equilibrium. A film with a net charge is self-repelling, requiring a larger mass of hydration water, leading to swelling. Such reversible, pH-induced LbL film swelling effects have been observed earlier in a simple pH-controlled electrostatic release system [40], and pH-controlled changes of the charge equilibrium were even used to control ion uptake in multilayer films [57]. In the present case, it is interesting to note that swelling occurs in basic, and de-swelling in acidic environment. Thus, it can be concluded that the as-prepared multilayer bears a negative excess charge, which is reduced upon protonation. 

In order to identify the role of additional contributions, a second control experiment with an alternating buffer change was performed on a clean, uncoated QCM-D sensor (see Supplementary Figure S11). Although even here a pH-dependent increase and decrease was found, the order of magnitude of the Δf change (ca. ±2 Hz) was much smaller than observed in the same experiment performed on the multilayer film. Such minor effect might be caused by slight differences between both liquids in density and viscosity, respectively [58]. Summarizing all these contributions, the significant Δf increase observed in Figure 6 displays the simultaneous occurrence of three different processes, i.e., the release of the guest, de-swelling of the multilayer film, and a negligible solvent effect.

**Figure 9:**
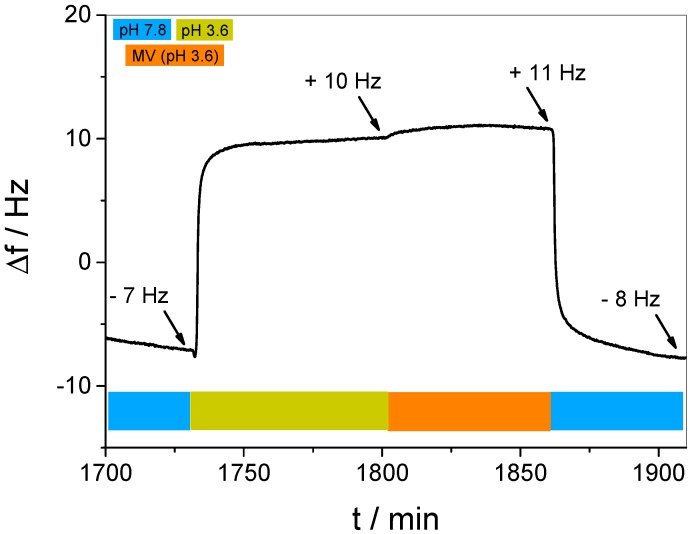
Δf change of the control experiment employing an acidic guest molecule solution (orange) to the multilayer films bearing WP6 in a fully protonated state (yellow).

Finally, we decided to deeper investigate the role of electrostatic interactions concerning the binding and release of the guest molecule. Therefore, we adjusted the unloaded multilayer film to acidic conditions to protonate WP6 (see Figure 9, highlighted in yellow), which yields the expected de-swelling. Subsequently, the film was exposed to MV solution (pH 3.6) in order to attempt binding to protonated WP6. As seen in Figure 9 (highlighted in orange), no significant change of Δf was monitored. This observation clearly demonstrates that the guest can only be bound with WP6 in the deprotonated state, therefore proving that the electrostatic interactions between host and guest is the main driving force for the host–guest complexation. In addition, the pH-responsiveness of the host–guest complexation can be concluded.

### 3.4. Quantification of Guest Surface Coverage by UV-Vis Spectra

The building blocks DAR and WP6 as well as the guest comprise a strong electronic absorbance in the UV-vis regime, respectively (see Supplementary Figures S1 and S7). Thus, we prepared dip-coated multilayer films (DAR/ WP6)_30_/DAR on quartz slides in order to determine the surface coverage of MV in the films by UV-vis spectroscopy. After preparation, the film spectrum on the quartz slides before crosslinking (see Supplementary Figure S12) agrees with that of DAR in solution (compare to Supplementary Figure S7), as expected, and therefore indicates a successful LbL assembly, as already described in our previous work [53].

The absorbance of DAR as the cationic building block of the film is clearly visible, having its maximum at 381 nm before photo-crosslinking (see Supplementary Figure S12, black line). In contrast, the absorbance of WP6, expected at ca. 290 nm (see Supplementary Figure S1), can only be weakly identified in Supplementary Figure S12. This observation corresponds to the formation of rather thin WP6 layers in the film, as indicated by the QCM-D data described above. The subsequent light irradiation decreases the DAR absorption band and therefore indicates a successful photo-crosslinking.

**Figure 10:**
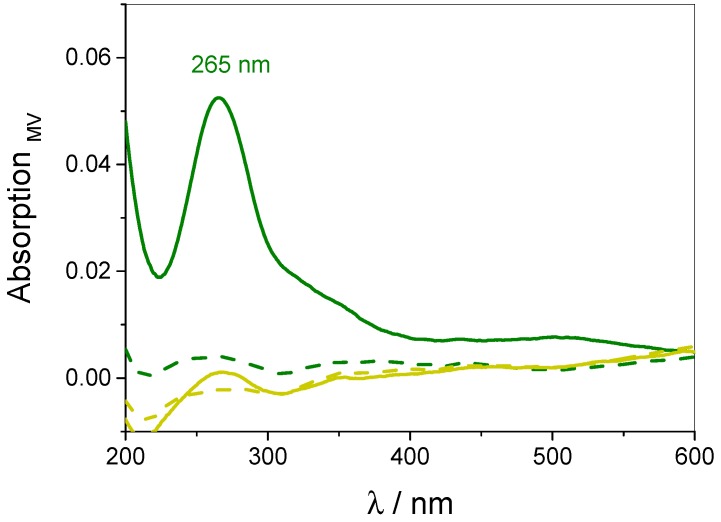
UV-vis spectra of MV after binding into a multilayer film at pH 7.8 (green continuous line) and subsequent release by pH 3.6 (green dotted line). The yellow spectra show analogue binding (continuous line) and release (dotted line) experiments performed at pH 3.6.

The WP6-containing films were saturated with MV in order to detect the surface coverage (Γ_MV_) of the incorporated guest molecule. Γ_MV_ was determined by measuring the absorption spectra of MV-saturated films referenced to an identical, but unloaded multilayer film (see Figure 10). Assuming that the incorporation of MV into the film does not influence the absorption properties of the components, the significant absorbance at 265 nm can be attributed to the incorporated MV and thus used in order to quantify the surface coverage Γ_MV_ with the help of a calibration line taken from MV solutions of different concentrations.

**Figure 11:**
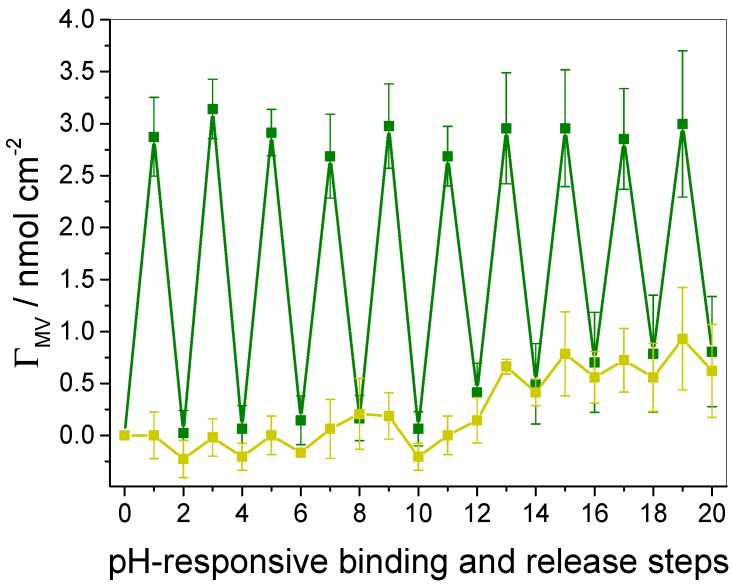
MV surface coverage per total film during ten binding and release cycles of MV into WP6-containing multilayer films. Γ_MV_ is given in dependence of the pH (green squares: binding at pH 7.8, release at pH 3.6; yellow squares: binding and release conducted at pH 3.6). Data are averaged over several samples.

Following this procedure over several samples, each undergoing ten pH-stimulated binding and release cycles, averaged data of Γ_MV_ was obtained and is given in Figure 11 (green squares). Γ_MV_ within a saturated film amounts to about 0.1 nmol cm^−2^ per layer. Please note that the decrease of the MV absorbance due to the complexation with WP6 is not regarded in the calculation of the surface coverage (compare Figure 3), therefore the corrected values are supposed to be slightly higher as indicated by Figure 11.

Within cycles one to five, identical amounts of MV were first bound and subsequently released from the film, thus reversibility as well as pH responsiveness of the binding event is again demonstrated. It is noteworthy that pH lowering allows the complete removal of the guest from the film without leaving any residuals. In conclusion, the WP6-containing multilayer films do not provide other sufficiently strong attractive interactions towards the guest, except of the desired host–guest interactions. This fact demonstrates a rare and outstanding property of these multilayer films compared to previous approaches, which were found to suffer from a decomposition of the binding sites, and moderate to enhanced unspecific binding of the guest due to secondary interactions [42,43,49,59].

The essential role of electrostatics was proven by a control experiment, wherein a series of identical films was exposed to an acidic MV solution. We once more conducted the binding and release procedure, but observed no incorporation of the guest into the films, as expected (see Figure 11, yellow squares). Therefore, such control experiments clearly identify the driving force of the host–guest complexation in WP6-containing multilayer films, in compliance with the data obtained from QCM-D experiments. However, for both experimental series, we noted a slight enrichment of the guest in the films after the fifth cycle (see green and yellow squares for steps 10 to 20 in Figure 11, respectively). This might be due to the multiple swelling and de-swelling events within the film as a consequence of the alternating pH changes, which might have caused structural rearrangements of the multilayer film and therefore lead to a low amount of irreversibly incorporated guest molecule. 

From the surface coverage of the guest molecule, conclusions can be drawn about the surface coverage of WP6: Assuming that the stoichiometry of the formed complex in the film is identical to that in solution, i.e., all WP6 cavities are accessible to MV binding, the surface coverage of WP6 is identical to that of MV, meaning that the average host coverage is 0.1 nmol cm^−2^ per bilayer. Taking our previous approaches employing cucurbituril into account [48,49,59,60], the CB[8]-based layer systems revealed a host coverage of 0.5 nmol cm^−2^ per bilayer, while classical SmiLbL films also yielded a binding site coverage of 0.1 nmol cm^−2^ per bilayer [40]. A potential explanation for the reduced host coverage of WP6 as compared to CB[8] might be the fact that WP6 was deposited as single molecule and without pre-association to a polyelectrolyte in order to reduce the content of non-binding and “inactive” material. Thus, the lower surface coverage of WP6 can probably be reasoned with the formation of rather thin anionic layers, as it was detected by QCM-D, due to the monomeric character of the building block and its high negative charge in the deprotonated state, which can yield low coverages due to electrostatic repulsion of neighboring host molecules.

## 4. Conclusions

In conclusion, we have proceeded in developing WP6-based polyelectrolyte multilayer release systems, and established the pH value as novel external stimulus for the control of the reversible complexation of a guest molecule. For this purpose, carboxylated WP6 was employed as negatively charged monomeric building block in the LbL assembly and then used as artificial binding site in the film. In this way, no second polymeric building block is required and thus the fraction of inactive polyelectrolyte material was reduced in comparison to previous host-containing films. The novel pH stimulus is based on the pH-dependent de-/protonation of the carboxylated rims of WP6. Since electrostatic attraction was determined as the main driving force of the host–guest complexation, the bound guest molecule could be easily released by a simple pH decrease, which leads to the protonated state of the WP6 and reduces the attractive host–guest interaction. Full reversibility of the pH-responsive binding and release over at least ten cycles, accompanied by a pH-dependent de/-swelling of the multilayer film, was clearly demonstrated. It is thus shown that the carboxylate groups can simultaneously accomplish three different function in the LbL release systems, as they (i) serve as charged groups to provide LbL assembly and (ii) partially react with DAR to accomplish crosslinking, and finally, (iii), the remaining fraction of unreacted carboxylic groups is sufficient to control guest binding via pH changes. Thus, we have therefore contributed to the promising field of host-containing polyelectrolyte multilayer films and future reports on further pillar[*n*]arene-containing multilayer films are anticipated due to the extensive modifiability of the host structure.

## Figures and Tables

**Table 1:** Thermodynamic parameters obtained from ITC (isothermal titration calorimetry) data fitted by a single site binding model for the titration of MV solution (1 mM) into WP6 solution (0.1 mM) at different pH values.

pH	k_a_/M^−1^	*n*	ΔH/kJ mol^−1^	ΔS/J mol^−1^ K^−1^
7.8	(1.53 ± 0.39) × 10^6^	0.92 ± 0.07	−18.2 ± 1.0	57.2 ± 1.6
4.8	(1.64 ± 0.42) × 10^5^	1.01 ± 0.02	−17.5 ± 1.0	40.9 ± 5.4

## Supplementary Materials

The following are available online at www.mdpi.com/2073-4360/9/12/719/s1, Figure S1: Absorbance spectra of carboxylated pillar[6]arene (WP6, 50 µM in 10 mM phosphate buffer); Figure S2: Absorbance spectra of the MV@WP6 complex (c = 250 μmol L^−1^) in 10 mM phosphate buffer (pH 7.8) showing the charge transfer band in the regime between 380 and 640 nm; Figure S3: Heating rate (upper plot) and binding isotherm (lower plot) of an isothermal titration calorimetry ITC experiment wherein a methylviologen dichloride (MV) solution (1 mmol L^−1^ in phosphate buffer, pH 7.8) was titrated into a WP6 solution (0.1 mmol L^−1^ in phosphate buffer, pH 7.8); Figure S4: Heating rate (upper plot) and binding isotherm (lower plot) of an ITC experiment wherein an MV solution (1 mmol L^−1^ in acetate buffer, pH 4.8) was titrated into a WP6 solution (0.1 mmol L^−1^ in acetate buffer, pH 4.8); Figure S5: Time-dependent change of the resonance frequency, Δf, for the nth overtone (n = 3, 5, 7, 9, 11) of the Layer-by-Layer assembly of a multilayer film (DAR/WP6)30/DAR; Figure S6: Absorbance spectra of (left plot, continuous line) a fresh DAR solution (50 μmol L^−1^, in 10 mM phosphate buffer, pH 7.8) before preparation and after 48h (left plot, dotted line). The chemical decomposition of DAR was followed by using the steady wavelength mode. Therefore, the fresh DAR solution was used in order to monitor the absorbance band of the diazo groups at 372 nm for 45h (right plot), giving the decrease in the absorbance value at a fixed wavelength with time; Figure S7: Absorbance spectra of DAR (50 μmol L^−1^) in 10 mM phosphate buffer (pH 7.8) after preparation (continuous line) and after 5 min VIS light irradiation (dotted line); Figure S8: Simultaneously detected change of dissipation (ΔD, red curve) during the LbL assembly of the multilayer film (DAR/WP6)_30_/DAR; Figure S9: Dissipative quartz crystal microbalance (QCM-D) control experiment: consecutive adsorption of 20 individual DAR layers without participation of WP6 as anionic building block; Figure S10: Four consecutive binding and release cycles of MV into a WP6-containing multilayer film (DAR/WP6)_30_/DAR. The binding of MV is represented by the green background, while the release performed by acetic buffer (pH 3.6) is highlighted in yellow. Blue displays the use of phosphate buffer (pH 7.8); Figure S11: Alternating change between phosphate buffer (pH 7.8) and acetic buffer (pH 3.8) on an uncoated QCM-D sensor. The procedure was repeated several times; Figure S12: UV-vis absorbance spectra of a multilayer film (DAR/WP6)_30_/DAR before (black line) and after photo-crosslinking (grey line).

## References

[1] Masson E., Ling X., Joseph R., Kyeremeh-Mensah L., Lu X. (2012). Cucurbituril chemistry: A tale of supramolecular success. RSC Adv..

[2] Barrow S.J., Kasera S., Rowland M.J., Del Barrio J., Scherman O.A. (2015). Cucurbituril-based molecular recognition. Chem. Rev..

[3] Yu G., Jie K., Huang F. (2015). Supramolecular amphiphiles based on host-guest molecular recognition motifs. Chem. Rev..

[4] Del Valle E.M.M. (2004). Cyclodextrins and their uses: A review. Process Biochem..

[5] Wang H., Shao N.M., Qiao S.N., Cheng Y.Y. (2012). Host-guest chemistry of dendrimer-cyclodextrin conjugates: Selective encapsulations of guests within dendrimer or cyclodextrin cavities revealed by NOE NMR techniques. J. Phys. Chem. B.

[6] Zhao J., Zhang Y.M., Sun H.L., Chang X.Y., Liu Y. (2014). Multistimuli-responsive supramolecular assembly of cucurbituril/cyclodextrin pairs with an azobenzene-containing bispyridinium guest. Chemistry.

[7] Isenbügel K., Gehrke Y., Ritter H. (2012). Photo-switchable behavior of azobenzene-dye-modified silica nanoparticles and their assembly with cyclodextrin derivatives. Macromol. Chem. Phys..

[8] Karakhanov E.A., Maximov A.L. (2010). Molecular imprinting technique for the design of cyclodextrin based materials and their application in catalysis. Curr. Org. Chem..

[9] Ravoo B.J., Darcy R. (2000). Cyclodextrin bilayer vesicles. Angew. Chem. Int. Ed..

[10] Behrend R., Meyer E., Rusche F.I. (1905). Ueber condensationsproducte aus glycoluril und formaldehyd. Eur. J. Org. Chem..

[11] Freeman W.A., Mock W.L., Shih N.Y. (1981). Cucurbituril. J. Am. Chem. Soc..

[12] Mock W.L., Shih N.Y. (1986). Structure and selectivity in host-guest complexes of cucurbituril. J. Org. Chem..

[13] Isaacs L. (2014). Stimuli responsive systems constructed using cucurbit[*N*]uril-type molecular containers. Acc. Chem. Res..

[14] Appel E.A., Biedermann F., Rauwald U., Jones S.T., Zayed J.M., Scherman O.A. (2010). Supramolecular cross-linked networks via host-guest complexation with cucurbit[8]uril. J. Am. Chem. Soc..

[15] Rauwald U., Scherman O.A. (2008). Supramolecular block copolymers with cucurbit[8]uril in water. Angew. Chem. Int. Ed..

[16] Ogoshi T., Yamagishi T. (2013). Pillararenes: Versatile synthetic receptors for supramolecular chemistry. Eur. J. Org. Chem..

[17] Ogoshi T., Kanai S., Fujinami S., Yamagishi T.A., Nakamoto Y. (2008). Para-bridged symmetrical pillar[5]arenes: Their lewis acid catalyzed synthesis and host-guest property. J. Am. Chem. Soc..

[18] Cao D.R., Meier H. (2015). Synthesis of pillar[6]arenes and their host-guest complexes. Synthesis-Stuttgart.

[19] Ogoshi T., Yamagishi T. (2013). New synthetic host pillararenes: Their synthesis and application to supramolecular materials. Bull. Chem. Soc. Jpn..

[20] Strutt N.L., Zhang H.C., Schneebeli S.T., Stoddart J.F. (2014). Functionalizing pillar[n]arenes. Acc. Chem. Res..

[21] Ogoshi T., Ueshima N., Sakakibara F., Yamagishi T., Haino T. (2014). Conversion from pillar[5]arene to pillar[6-15]arenes by ring expansion and encapsulation of c-60 by pillar[n]arenes with nanosize cavities. Org. Lett..

[22] Ogoshi T., Hashizume M., Yamagishi T.A., Nakamoto Y. (2010). Synthesis, conformational and host-guest properties of water-soluble pillar[5]arene. Chem. Commun..

[23] Tan L.L., Yang Y.W. (2015). Molecular recognition and self-assembly of pillarenes. J. Incl. Phenom. Macrocycl. Chem..

[24] Hu X.Y., Jia K.K., Cao Y., Li Y., Qin S., Zhou F., Lin C., Zhang D.M., Wang L.Y. (2015). Dual photo- and ph-responsive supramolecular nanocarriers based on water-soluble pillar[6]arene and different azobenzene derivatives for intracellular anticancer drug delivery. Chem. Eur. J..

[25] Zhou Q.Z., Jiang H.J., Chen R., Qiu F.L., Dai G.L., Han D.M. (2014). A triply-responsive pillar[6]arene-based supramolecular amphiphile for tunable formation of vesicles and controlled release. Chem. Commun..

[26] Cao Y., Hu X.Y., Li Y., Zou X.C., Xiong S.H., Lin C., Shen Y.Z., Wang L.Y. (2014). Multistimuli-responsive supramolecular vesicles based on water-soluble pillar[6]arene and saint complexation for controllable drug release. J. Am. Chem. Soc..

[27] Yu G.C., Zhou X.R., Zhang Z.B., Han C.Y., Mao Z.W., Gao C.Y., Huang F.H. (2012). Pillar[6]arene/paraquat molecular recognition in water: High binding strength, ph-responsiveness, and application in controllable self-assembly, controlled release, and treatment of paraquat poisoning. J. Am. Chem. Soc..

[28] Wang P., Yao Y., Xue M. (2014). A novel fluorescent probe for detecting paraquat and cyanide in water based on pillar[5]arene/10-methylacridinium iodide molecular recognition. Chem. Commun..

[29] Ogoshi T., Kida K., Yamagishi T. (2012). Photoreversible switching of the lower critical solution temperature in a photoresponsive host-guest system of pillar[6]arene with triethylene oxide substituents and an azobenzene derivative. J. Am. Chem. Soc..

[30] Li Z.T., Yang J., Yu G.C., He J.M., Abliz Z., Huang F.H. (2014). Synthesis of a water-soluble pillar[9]arene and its ph-responsive binding to paraquat. Chem. Commun..

[31] Chen H.Q., Jia X.S., Li C.J. (2015). A pillar[6]arene-[2]pseudorotaxane based ph-sensitive molecular switch. Chin. J. Chem..

[32] Decher G., Hong J.D., Schmitt J. (1992). Buildup of ultrathin multilayer films by a self-assembly process: 3. Consecutively alternating adsorption of anionic and cationic polyelectrolytes on charged surfaces. Thin Solid Films.

[33] Decher G. (1997). Fuzzy nanoassemblies: Toward layered polymeric multicomposites. Science.

[34] Jaber J.A., Schlenoff J. (2006). Recent developments in the properties and applications of polyelectrolyte multilayers. Curr. Opin. Colloid Interface Sci..

[35] Klitzing R.V. (2006). Internal structure of polyelectrolyte multilayer assemblies. Phys. Chem. Chem. Phys..

[36] Xu H.P., Schönhoff M., Zhang X. (2012). Unconventional layer-by-layer assembly: Surface molecular imprinting and its applications. Small.

[37] Wulff G. (1995). Molecular imprinting in cross-linked materials with the aid of molecular templates—A way towards artificial antibodies. Angew. Chem. Int. Ed..

[38] Wulff G. (1996). Molecular imprinting in crosslinked polymers—The role of the binding sites. Mol. Cryst. Liq. Cryst..

[39] Shi F., Liu Z., Wu G.L., Zhang M., Chen H., Wang Z.Q., Zhang X., Willner I. (2007). Surface imprinting in layer-by-layer nanostructured films. Adv. Funct. Mater..

[40] Gauczinski J., Liu Z., Zhang X., Schönhoff M. (2010). Mechanism of surface molecular imprinting in polyelectrolyte multilayers. Langmuir.

[41] Zhou Y., Cheng M., Zhu X., Zhang Y., An Q., Shi F. (2013). A facile method to prepare molecularly imprinted layer-by-layer nanostructured multilayers using postinfiltration and a subsequent photo-cross-linking strategy. ACS Appl. Mater. Interfaces.

[42] Niu J., Shi F., Liu Z., Wang Z.Q., Zhang X. (2007). Reversible disulfide cross-linking in layer-by-layer films: Preassembly enhanced loading and ph/reductant dually controllable release. Langmuir.

[43] Guan G., Liu R., Wu M., Li Z., Liu B., Wang Z., Gao D., Zhang Z. (2009). Protein-building molecular recognition sites by layer-by-layer molecular imprinting on colloidal particles. Analyst.

[44] Zhang J.W., Liu Y.L., Wu G.L., Schönhoff M., Zhang X. (2011). Bolaform supramolecular amphiphiles as a novel concept for the buildup of surface-imprinted films. Langmuir.

[45] Niu J., Liu Z., Fu L., Shi F., Ma H., Ozaki Y., Zhang X. (2008). Surface-imprinted nanostructured layer-by-layer film for molecular recognition of theophylline derivatives. Langmuir.

[46] Gauczinski J., Liu Z.H., Zhang X., Schönhoff M. (2012). Surface molecular imprinting in layer-by-layer films on silica particles. Langmuir.

[47] Zhang J.W., Liu Y.L., Yuan B., Wang Z.Q., Schönhoff M., Zhang X. (2012). Multilayer films with nanocontainers: Redox-controlled reversible encapsulation of guest molecules. Chem. Eur. J..

[48] Nicolas H., Yuan B., Zhang X., Schönhoff M. (2016). Cucurbit[8]uril-containing multilayer films for the photocontrolled binding and release of a guest molecule. Langmuir.

[49] Nicolas H., Yuan B., Zhang J., Zhang X., Schönhoff M. (2015). Cucurbit[8]uril as nanocontainer in a polyelectrolyte multilayer film: A quantitative and kinetic study of guest uptake. Langmuir.

[50] Bian Q., Jin M.M., Chen S., Xu L.P., Wang S.T., Wang G.J. (2017). Visible-light-responsive polymeric multilayers for trapping and release of cargoes via host-guest interactions. Polym. Chem..

[51] Xuan H.Y., Ren J.Y., Zhang J.H., Ge L.Q. (2017). Novel highly-flexible, acid-resistant and self-healing host-guest transparent multilayer films. Appl. Surf. Sci..

[52] Xu G., Pranantyo D., Xu L.Q., Neoh K.G., Kang E.T., Teo S.L.M. (2016). Antifouling, antimicrobial, and antibiocorrosion multilayer coatings assembled by layer-by-layer deposition. Involving host-guest interaction. Ind. Eng. Chem. Res..

[53] Yuan B., Xu J.F., Sun C.L., Nicolas H., Schönhoff M., Yang Q.Z., Zhang X. (2016). Pillar[6]arene containing multilayer films: Reversible uptake and release of guest molecules with methyl viologen moieties. ACS Appl. Mater. Interfaces.

[54] Sun J.Q., Wu T., Liu F., Wang Z.Q., Zhang X., Shen J.C. (2000). Covalently attached multilayer assemblies by sequential adsorption of polycationic diazo-resins and polyanionic poly(acrylic acid). Langmuir.

[55] Morgan G.T., Alcock M. (1909). The colour and constitution of diazonium salts. Part I. J. Chem. Soc..

[56] Ostendorf A., Cramer C., Decher G., Schönhoff M. (2015). Humidity-tunable electronic conductivity of polyelectrolyte multilayers containing gold nanoparticles. J. Phys. Chem. C.

[57] Parveen N., Schönhoff M. (2017). Quantifying and controlling the cation uptake upon hydrated ionic liquid-induced swelling of polyelectrolyte multilayers. Soft Matter.

[58] Nomura T., Okuhara M. (1982). Frequency-shifts of piezoelectric quartz crystals immersed in organic liquids. Anal. Chim. Acta.

[59] Nicolas H., Yuan B., Zhang J.W., Zhang X., Schönhoff M. (2017). Correction to “Cucurbit[8]uril as nanocontainer in a polyelectrolyte multilayer film: A quantitative and kinetic study of guest uptake”. Langmuir.

[60] Nicolas H., Yuan B., Zhang X., Schönhoff M. (2017). Correction to “Cucurbit[8]uril-containing multilayer films for the photocontrolled binding and release of a guest molecule”. Langmuir.

