# Supplementary material for: pH-Responsive Host–Guest Complexation in Pillar[6]arene-Containing Polyelectrolyte Multilayer Films
Source: Polymers (Basel). 2017 Dec 16;9(12):719. doi: 10.3390/polym9120719 (PMC6418545; doi:10.3390/polym9120719)
Supplement: Supplementary file 1 [file polymers-09-00719-s001.pdf]

polymers

MDPI

pH-Responsive Host-Guest Complexation in Pillar[6]arene-Containing Polyelectrolyte Multilayer Films

Henning Nicolas [1], Bin Yuan [2], Jiangfei Xu[2], Xi Zhang[2] and Monika Schönhoff [1,*]

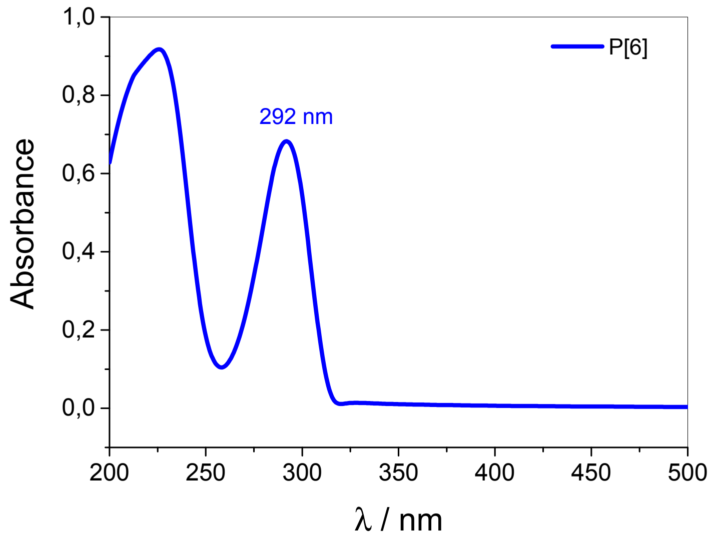

Figure S1. Absorbance spectra of carboxylated pillar[6]arene (WP6, 50 μM in 10 mM phosphate buffer).

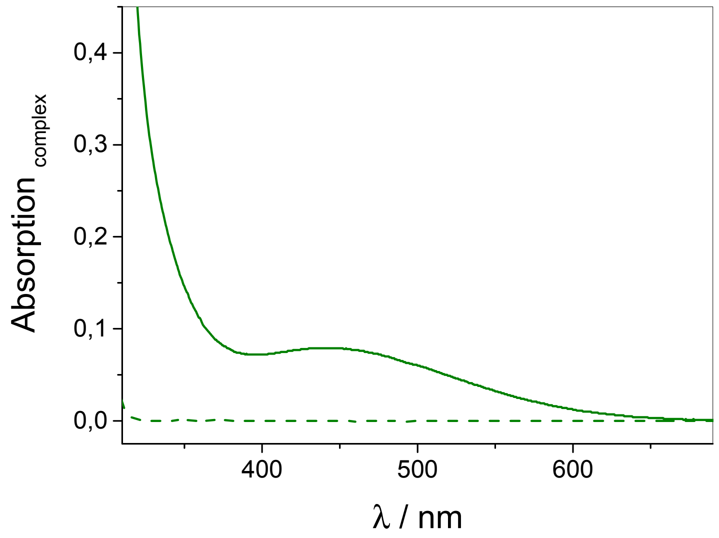

Figure S2. Absorbance spectra of the MV@WP6 complex (c = 250 μmol L^{-1}) in 10 mM phosphate buffer (pH 7.8) showing the charge transfer band in the regime between 380 and 640 nm.

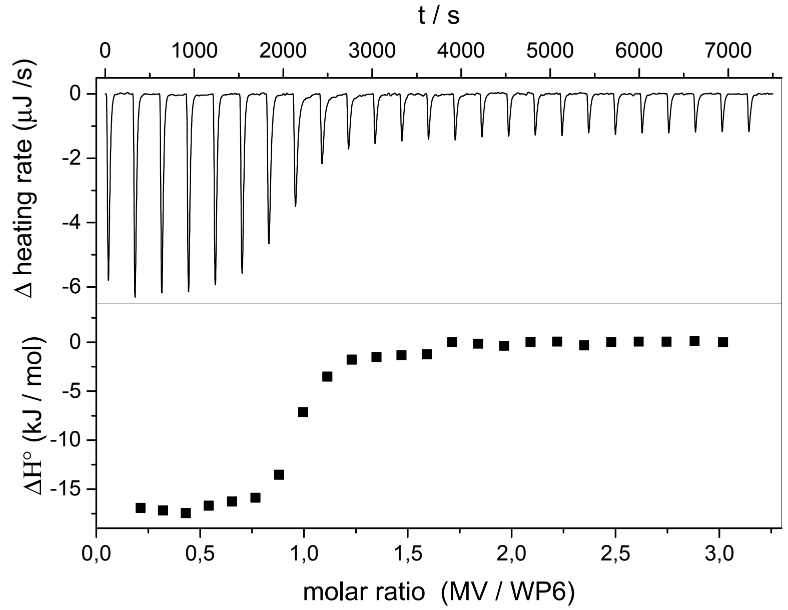

Figure S3. Heating rate (upper plot) and binding isotherm (lower plot) of an isothermal titration calorimetry ITC experiment wherein a methylviologen dichloride (MV) solution (1 mmol L^{-1} in phosphate buffer, pH 7.8) was titrated into a WP6 solution (0.1 mmol L^{-1} in phosphate buffer, pH 7.8).

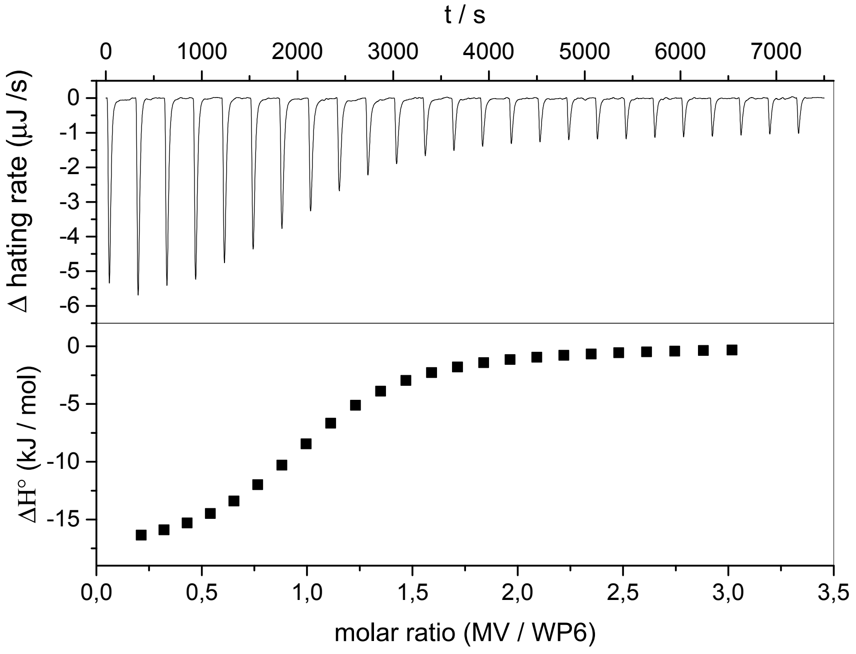

Figure S4. Heating rate (upper plot) and binding isotherm (lower plot) of an ITC experiment wherein an MV solution (1 mmol L^{-1} in acetate buffer, pH 4.8) was titrated into a WP6 solution (0.1 mmol L^{-1} in acetate buffer, pH 4.8).

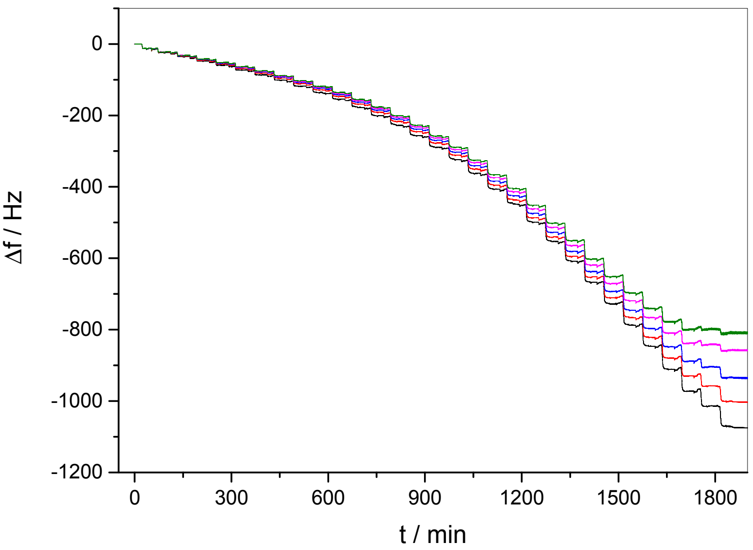

Figure S5. Time-dependent change of the resonance frequency, Δf, for the nth overtone (n = 3, 5, 7, 9, 11) of the Layer-by-Layer assembly of a multilayer film $(DAR/WP6)_{30}/DAR$.

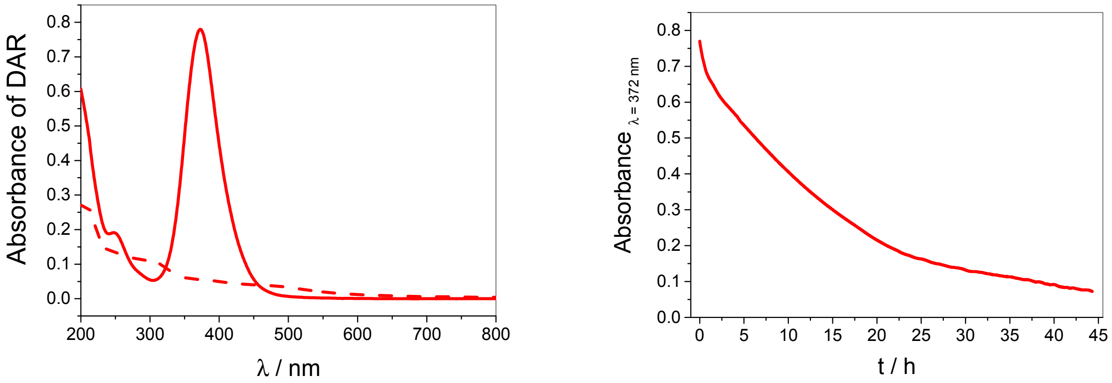

Figure S6. Absorbance spectra of (left plot, continuous line) a fresh DAR solution (50 µmol L^{-1}, in 10 mM phosphate buffer, pH 7.8) before preparation and after 48h (left plot, dotted line). The chemical decomposition of DAR was followed by using the steady wavelength mode. Therefore, the fresh DAR solution was used in order to monitor the absorbance band of the diazo groups at 372 nm for 45h (right plot), giving the decrease in the absorbance value at a fixed wavelength with time.

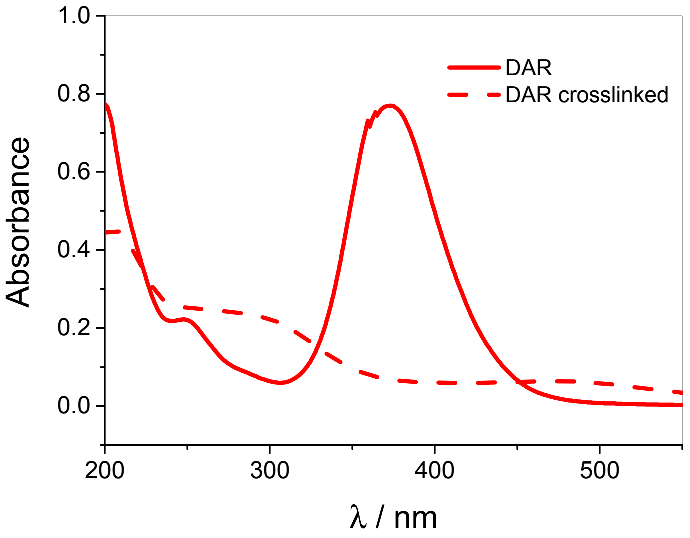

Figure S7. Absorbance spectra of DAR (50 μmol L^{-1}) in 10 mM phosphate buffer (pH 7.8) after preparation (continuous line) and after 5 min VIS light irradiation (dotted line).

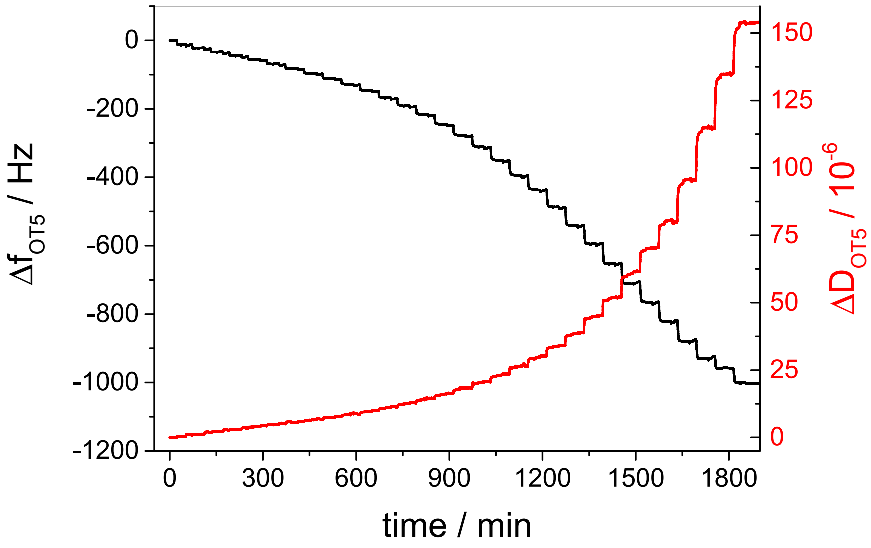

Figure S8. Simultaneously detected change of dissipation (ΔD, red curve) during the LbL assembly of the multilayer film $(DAR/WP6)_{30}/DAR$.

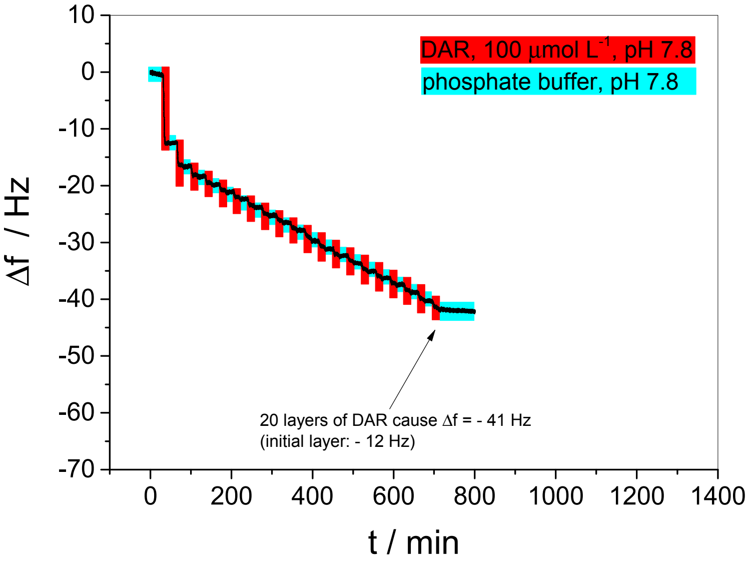

Figure S9. Dissipative quartz crystal microbalance (QCM-D) control experiment: consecutive adsorption of 20 individual DAR layers without participation of WP6 as anionic building block.

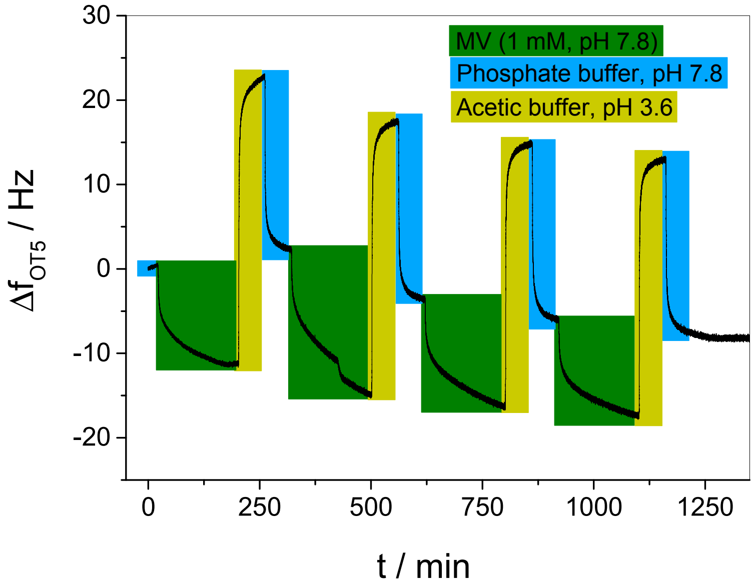

Figure S10. Four consecutive binding and release cycles of MV into a WP6-containing multilayer film $(DAR/WP6)_{30}/DAR$. The binding of MV is represented by the green background, while the release performed by acetic buffer (pH 3.6) is highlighted in yellow. Blue displays the use of phosphate buffer (pH 7.8).

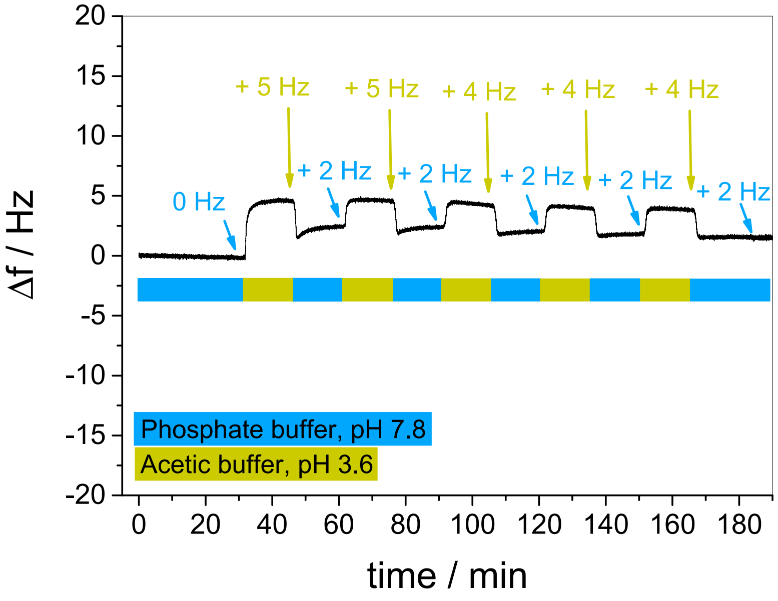

Figure S11. Alternating change between phosphate buffer (pH 7.8) and acetic buffer (pH 3.8) on an uncoated QCM-D sensor. The procedure was repeated several times.

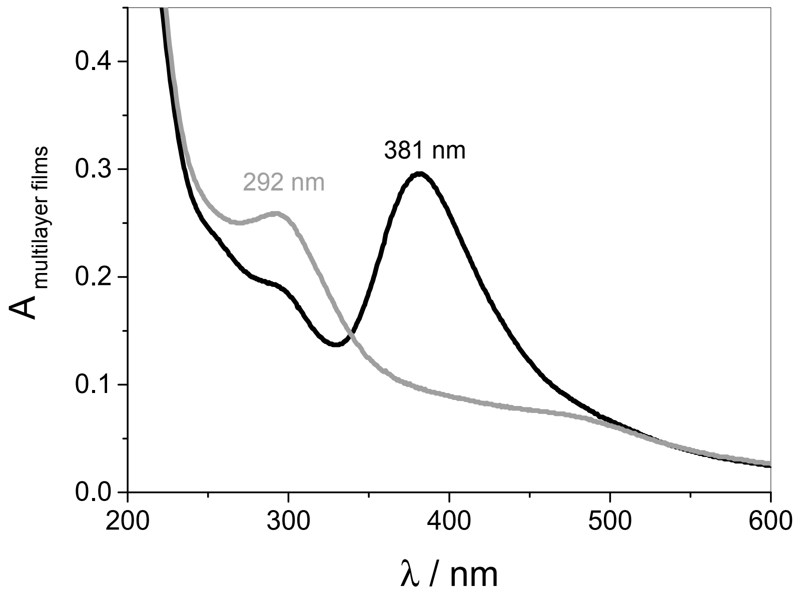

Figure S12. UV-vis absorbance spectra of a multilayer film $(DAR/WP6)_{30}/DAR$ before (black line) and after photo-crosslinking (grey line).
